# Neither Threat of Shock nor Acute Psychosocial Stress Affects Ambiguity Attitudes

**DOI:** 10.1007/s42761-022-00109-6

**Published:** 2022-04-07

**Authors:** Deshawn Chatman Sambrano, Arlene Lormestoire, Candace Raio, Paul Glimcher, Elizabeth A. Phelps

**Affiliations:** 1 Department of Psychology, Harvard University, Cambridge, MA USA; 2 Department of Psychology, Princeton University, Princeton, NJ USA; 3 Neuroscience Institute, New York University Grossman School of Medicine, New York, NY USA

**Keywords:** Emotion and decision making, Ambiguity aversion, Uncertainty

## Abstract

Decisions under uncertainty can be differentiated into two classes: risky, which has known probabilistic outcomes, and ambiguous, which has unknown probabilistic outcomes. Across a variety of types of decisions, people find ambiguity extremely aversive, subjectively more aversive than risk. It has been shown that the transient sympathetic arousal response to a choice predicts decisions under ambiguity but not risk, and that lifetime stress uniquely predicts attitudes toward ambiguity. Building on these findings, this study explored whether we could bias ambiguity and risk preferences with an arousal or acute stress manipulation that is incidental to the choice in two independent experiments. One experiment induced sympathetic arousal with an anticipatory threat paradigm, and the other manipulated incidental acute stress via a psychosocial stressor. The efficacy of the manipulations was confirmed via pupil dilation and salivary cortisol, respectively. Participants made choices between a guaranteed $5 option and a lottery with either a known (risky) or unknown (ambiguous) probabilistic outcome. Consistent with previous findings, participants were more averse to a given level of ambiguity than to a numerically equal level of risk. However, in contrast to our hypothesis, we found no evidence that transient arousal or acute stress that is incidental to the choice biases ambiguity preferences.

Imagine you are at the doctor’s office, recently diagnosed with a life-threatening disease, and you have two treatment options. The traditional treatment offers a 50% success rate, while a newly designed drug has a success rate somewhere between 30% and 80%. Which drug would you take? What if the situation was less stressful, say, the treatment was to make your skin appear more youthful? Would your decision change?

The traditional drug represents a risky option in which the probabilities of success are known, whereas the efficacy of the new drug is ambiguous because the outcome’s probabilities are unknown. Across a myriad of decision types, people find ambiguity more aversive than risk to the point that they will choose a risky option even when the ambiguous choice has a higher expected value (Ellsberg, 1961; Slovic & Tversky, 1974). This bias toward the risky options can have negative consequences when, as in the treatment example, the ambiguous option has a higher expected success rate. Without prior knowledge, all percentage chances between 30% and 80% are equally likely and when integrated over, one should expect the average success rate to be 55% (five points higher than the traditional treatment). Ambiguity aversion’s potential negative consequences are not only apparent with health decisions (Han et al., 2009) but extend across decision types, including social (Li et al., 2020) and career decisions (Xu, 2020). Given these potential negative consequences of ambiguity aversion, the goal of this study is to examine how the affective states arousal and stress affect ambiguity attitudes.

Physiological arousal, one component of emotion (Scherer, 2005), has been shown to be involved in the processing of ambiguity. Feldmanhall et al. (2016) measured phasic physiological arousal with skin conductance response, while participants chose between a guaranteed amount and a lottery that was either risky or ambiguous. The authors found that skin conductance predicted whether participants would choose the ambiguous lottery but had no predictive utility for decision making over risky lotteries. Another study, which used the same paradigm, found that increased blood oxygenation level-dependent (BOLD) signal in the amygdala, a brain region that often responds to emotional events and stimuli, was uniquely observed to ambiguous, but not risky, choices (Levy et al. 2010). These initial findings suggest that an emotional reaction may play a role in differentiating preferences for these types of uncertainty.

In these studies, the emotional response is *integral* to the choice, in that it is the choice itself driving arousal or amygdala activation (Lerner et al., 2015; Phelps et al., 2014). Integral emotions are theorized to provide information (Schwarz, 2012), serve as a guide (Lerner et al., 2015), or signal a choice option’s subjective value (Phelps et al., 2014) to help one make better decisions. Other times our experienced emotions are *incidental* to the choice itself, in that an unrelated affective state impacts the decision. Principally, there is no normative reason for incidental affective states to influence our choices because the affective state is unrelated to the choice, but previous studies demonstrate that they do (see Lerner et al., 2015; Loewenstein & Lerner, 2003 and Phelps et al., 2014 for a review). A recent example of an incidental affective state differentially impacting assessments of risk and ambiguity is a study showing that higher levels of lifetime stress correlate with greater ambiguity aversion but were unrelated to risk attitudes (Raio et al., 2021). Raio et al. (2021) hypothesized that a history of stressful events may shape an individual’s subjective assessment of ambiguous options causing them to avoid ambiguous choices.

In the present study, we build on these previous studies in two ways. First, we assess the impact of incidental arousal and acute (as opposed to lifetime) stress on ambiguity preferences. Second, these previous studies are correlational; we will therefore test whether there is a causal relationship between arousal, stress, and ambiguity aversion with experimental stress manipulations.

Our bodies’ physiological stress response is modulated by the sympathetic-adrenomedullary (SAM) and the hypothalamic–pituitary–adrenal (HPA) axis. Although both SAM and HPA axis responses are components of the stress response, for clarity we will refer to the SAM response as *arousal* and HPA axis response as *stress*. The SAM response is rapid and can be measured almost immediately through skin conductance, pupil dilation, heart rate, as well as through free salivary α-amylase 10 to 12 min after the stressor (Koh et al., 2014). In contrast, the HPA axis, commonly measured by salivary cortisol, has a relatively slower peak response typically about 30 to 60 min after the stressful event (Kirschbaum et al., 1993; Qi et al., 2016). In addition to their variable time scales SAM and HPA axis responses can be differentiated with experimental manipulations. For example, threat of shock manipulations (Maruyama et al., 2012; Torrisi et al., 2016) and viewing emotional images (van Stegeren et al., 2008; Wang et al., 2018) reliably increase physiological measures of arousal and salivary α-amylase, but not cortisol. In contrast, stressors like the Trier Social Stress Test (TSST; Maruyama et al., 2012) and the cold pressor task (CPT; van Stegeren et al., 2008) reliably increase both neuroendocrine markers. Given the differences between the two neuroendocrine systems and the two ways in which emotion can affect decision making, we can identify the interactions between arousal, acute stress, and decision making as well as which component of the incidental arousal/stress response, if any, impacts the decision making process.

The current study explores whether physiological arousal and acute stress incidental to the choice biases ambiguity preferences via two independent experiments dissociating the two neuroendocrine stress responses. One study induced an arousal response via an anticipatory threat manipulation, and the other manipulated incidental stress via an acute psychosocial stressor. If incidental arousal or acute stress is a causal mechanism for ambiguity aversion, then enhancing arousal or stress should alter ambiguity aversion. Alternatively, if incidental arousal or stress and ambiguity aversion are not linked, then there should be no differences in attitudes between the two contexts. Importantly, consistent with previous literature examining integral arousal (FeldmanHall et al., 2016) and cumulative stress over the lifespan (Raio et al., 2021), we expect incidental arousal and stress will have no impact on risk attitudes. Finally, by using two different stress manipulations, we can differentiate the influence of the physiological arousal component of the SAM response on ambiguity attitudes from the HPA axis stress responses.

## Experiment 1: Threat of Shock

### Method

#### Participants

Fifty-eight individuals participated in a lottery task that incorporated both risky and ambiguous lotteries (Levy et al., 2010; FeldmanHall et al., 2016) in the laboratory. Participants received $20 per hour with a bonus of up to $66 based on the performance in the lottery task. The sample size was estimated based on a prior study using the same task demonstrating a relationship between integral arousal and ambiguity aversion (FeldmanHall et al., 2016). Participants were excluded if they were not fluent in English, had a prior history of mental illness, or had participated in a study in which mild shocks were presented in the previous 12 months. All participants were informed prior to the study that it would involve the administration of mild electric shocks to the wrist. The study was approved by the New York University Institutional Review Board. Participants were excluded due to poor model fit demonstrated by outlier cases of pseudo *R*^2^ (*N* = 6) and lack of choice variability (e.g., always choosing the guaranteed amount) which made it estimating attitudes impossible (*N* = 1) leaving a total of 51 participants which were included for analyses of model parameters (67% Female; *M*_Age_ = 21.33, *SD*_Age_ = 3.33) participants. The datasets generated during and/or analyzed during the current study are available in the Open Science Framework repository, https://osf.io/n6em2/.

#### Measures

We employed a lottery based decision making task that has been previously demonstrated to disentangle risk and ambiguity attitudes (Levy et al., 2010; FeldmanHall et al., 2016; Raio et al., 2021). Participants were asked on each trial to decide whether they preferred a guaranteed amount of $5 or to play a lottery to potentially earn more money. Lotteries contained 100 red and blue chips, and the proportion of red to blue chips varied on each trial. On risky trials, participants were informed of the full distribution of chips (e.g., there are exactly 75 red chips and 25 blue chips). On ambiguous trials, there was a gray bar obscuring the full distribution, and participants were only aware of a range (e.g., there were at least 25 red chips and at least 25 blue chips, but the remaining 50 were unknown). The chips the gray bar obscured could be all red, all blue, or some combination of the two. In total, there were three risky lotteries (with winning probabilities of 25%, 50%, and 75%), and three ambiguous lotteries (with winning probabilities ranging between 38%–62%, 25%–75%, and 13%–87%). On each trial, there was a variable winning amount ($5–$66) associated with one chip color; the losing chip color was worth $0. The participant was offered a choice between playing the lottery or taking a certain amount of $5. The winning amount, winning probability, and chip color were counterbalanced for each subject such that the expected winning amount between the chip colors was equated over the course of the experiment.

By having participants directly make choices between a certain option and a risky lottery, we were able to estimate participants attitudes toward risk. Since participants saw the same winning amounts for the risky and ambiguity lotteries, any differences in the average number of trials the lottery was chosen between ambiguous trials and risky trials are evidence that ambiguity affected their choices, over and above that of risk. Importantly, participants were instructed that all ambiguous and risky lotteries shown were representations of one of the six physical urns shown to participants during instruction. Thus, any expectation from participants that the experimenter manipulated the urns was mitigated by the fact that they saw the same urn multiple times, some with the blue chips winning and some with the red chips winning, equated on expected value.

Before the instructions of the task, participants indicated their anxiety on a 0 (none at all) to 100 (intense) scale. Next, participants completed a lottery-based decision making task in order to estimate their attitudes on risk and ambiguity. Once participants completed the lottery task, they were again asked to rate their current anxiety. Following this rating, participants retrospectively indicated their anxiety levels during the threat and safe blocks separately. Finally, they completed a series of surveys including: the Positive Affect Negative Affect Scale (PANAS; Watson et al., 1988), the State Trait Anxiety Inventory (STAI; Spielberger et al., 1983), and the Intolerance of Uncertainty (IoU) scale (Carleton et al., 2007). These scales were completed to conduct exploratory analyses examining any moderating effects of these trait variables.

#### Pupil Dilation

We measured pupil dilation using an EyeLink 1000 Plus eye-tracker (www.sr-research.com) as a proxy for sympathetic arousal. The primary advantage for pupil dilation over skin conductance is the speed at which trials can progress. A typical skin conductance response takes about 8 s to recover after stimulus onset. In contrast, one can obtain a pupil response in 1 to 3 s. Given that participants completed 240 trials in addition to instructions, practice, calibration, and surveys, this cut the total experiment time roughly in half. Each trial had a baseline measure of 1 s recorded during the ITI. Trial-by-trial pupil dilation was measured as the average dilation during the lottery screen minus the 1-s baseline. All five colors used in this experiment were selected to maintain constant luminance on a 21.5-inch dell monitor. On average, each color measured 10.34 cd/m^2^ (range 9.93–10.67 cd/m^2^). Data preprocessing was done following the guidelines illustrated in Kret and Sjak-Shie (2019). Pre-trial baseline was required for all analyses; thus, trials in which a shock was administered during the ITI were excluded for pupil analysis in addition to the two lottery shock trials.

#### Procedures

After the initial emotion questionnaire, participants were instructed on the lottery task and given 40 practice trials to get used to the task. Once participants were comfortable with the task and indicated they had no further questions, we calibrated the electric shocks and the eye tracker. Participants received a mild electric shock to the wrist and rated their pain on a scale from zero (no pain at all) to nine (intense pain). We used a staircase method to ensure that the shock was uncomfortable (6 or 7), but not painful. An Astro-med Grass Pulse Stimulator (model SD9) was used to administer the electric shock.

**Fig. 1:**
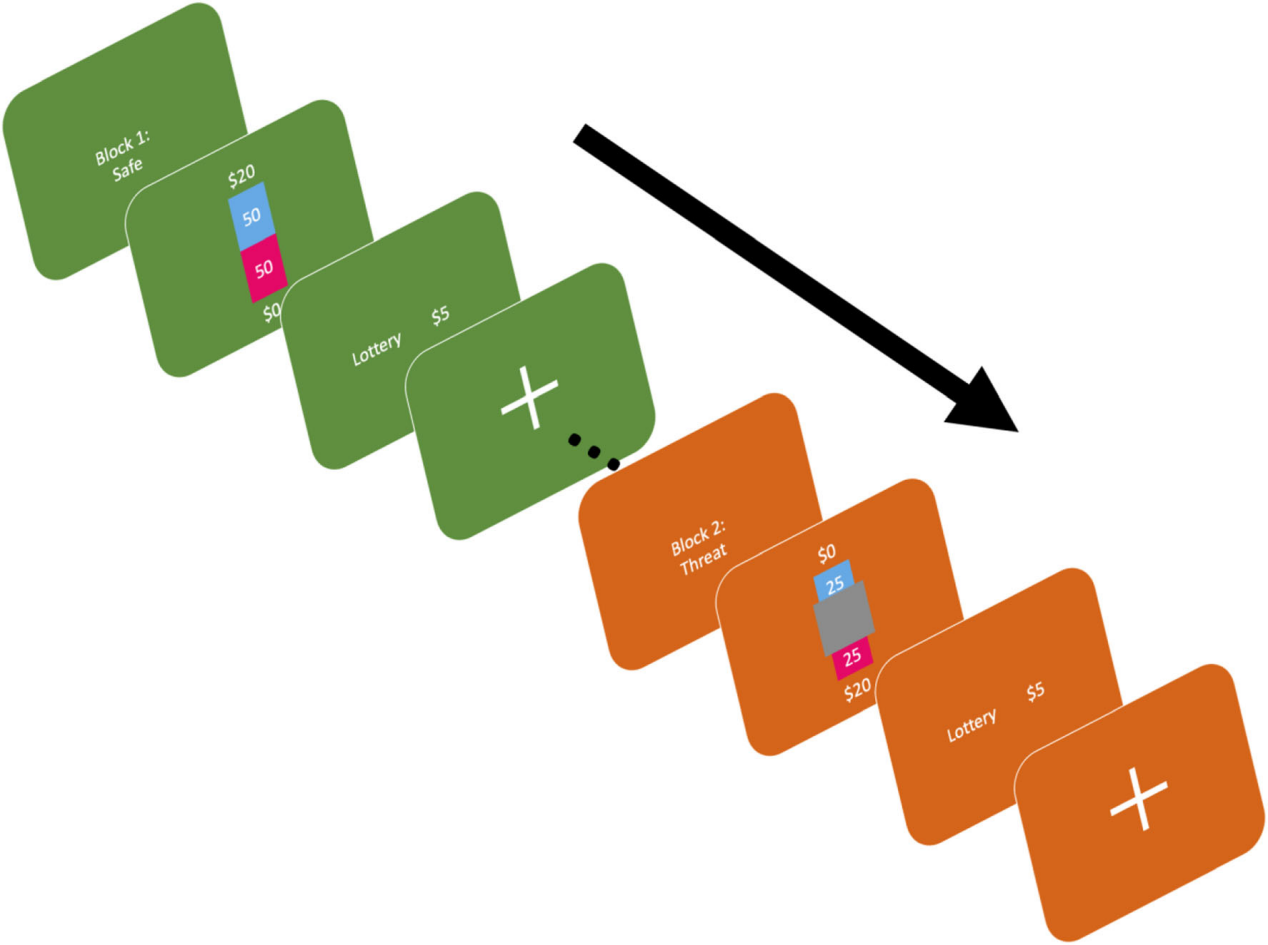
Trial and block structure. Blocks began with a screen to indicate context (threat or safe). The background color served as a reminder throughout the block. Each trial started with the lottery displayed for 4 s. Next, the participants had 1.5 s to make a selection between the “lottery” and guaranteed “$5” after which a rectangle appeared around their choice for 0.5 s. Between each trial participants saw a fixation cross with a variable ITI

For the main task, there were 16 blocks each consisting of 15 trials that alternated between a safe context and a context where they were under threat of shock (block order was randomized across subjects). Within any given block, the risky and ambiguous trials were random; however, participants saw all risky and all ambiguous trials under both contexts. Participants were informed that shocks could only occur during a threat block and that the shocks were random and not tied to their choices in any way. At the beginning of each block, we displayed the block number and the block type. The background screen was colored either green or brown indicating a safe or threat block (color and block type counterbalanced across subjects). The background color remained throughout the duration of the block to help remind participants which context they were in. Each trial began with a lottery in the center of the screen for 4 s followed by the words “Lottery” and “$5” on the screen (word order was counterbalanced across subjects) for 1.5 s. Participants had 5.5 s to decide on each trial. For the first 4 s participants viewed the lottery on screen, which was followed by a 1.5-s interval to indicate their choice with a left or right arrow key. If they did not indicate a response during the 1.5-s response window, no choice was recorded and that trial would not be eligible for the bonus if randomly selected at the end of the experiment. Following their choice, the screen remained for another .5 s with a rectangle around their choice to serve as confirmation that their choice was selected. This was followed by a variable intertrial-interval (ITI; 1.75–2.25 s; see Figure 1) with a fixation cross. Blocks lasted, on average, 120 s each. All participants received 7 shocks in total in a pseudorandom order. They could receive multiple shocks in a single block or none at all. Additionally, the shock could come during the ITI (*N* = 5) or during the presentation of a lottery (*N* = 2). Trials where shocks were administered during the lottery presentation were excluded from analyses. Block type (safe vs. threat) was counterbalanced across subjects.

#### Quantifying Risk and Ambiguity Attitudes

Detecting differences in the average choices between risky and ambiguous lotteries yields some evidence for ambiguity aversion; it has limitations. First, it is a relatively crude measure that may not pick up on subtle differences between uncertainty types. Second, it yields no concrete estimates of expected behavior outside the specific ambiguity levels selected in the experiment. To solve both problems, we used a computational model utilized in previous studies (Levy et al., 2010; FeldmanHall et al., 2016) to determine the subjective value of the choice options using a power function (Kahneman & Tversky, 1979) that takes into account the effect of ambiguity (Gilboa & Schmeidler, 1989),

$$ SV\left(p,A,v\right)=\left(p-\beta \frac{A}{2}\right)\times {v}^{\alpha } $$

using the probability of winning (*p*), level of ambiguity (*A*; the proportion of chips of unknown color), and winning amount (*v*) as arguments to the function. Additionally, there were two individual specific parameters to measure attitudes toward risk (*α*) and attitudes toward ambiguity (*β*). Crucially, the model makes more precise predictions (compared to raw choice data) and yields a parameter estimate that can indicate how averse one person is as well as predict how that person would behave in a context with ambiguity other than the three specifically selected for this experiment.

To provide a general intuition of the model, we illustrate the following example. A *β* of zero would cancel out the ambiguity term ($-\upbeta \frac{A}{2}$) leaving you with *p* × *v*^α^ (thus, someone with a *β* would not be affected by any amount of ambiguity). However, as *β* increases, the overall function (SV) decreases (i.e., ambiguity aversion, they avoid the lottery because it has less value) because of the negative coefficient in front of *β*. In contrast, as *β* decreases, the SV of the lottery increases (i.e., ambiguity seeking). A similar example can be done for *α* (for simplicity it is easiest to assume *A* = 0 for this example), where 1 is the neutral point, *α* > 1 is risk seeking, and *α* < 1 are risk averse.

Participants’ subjective value of the lottery (*SV*_L_) and the reference (*SV*_*R*_; substituting the following values: *p* = 1, *A* = 0, *v* = 5) were used to obtain a probability of choosing the lottery via a standard probabilistic choice function with an individual specific inverse temperature term (*γ*):

$$ p\left( Choose\ Lottery\right)=\frac{1}{1+{e}^{\upgamma \left({SV}_R-{SV}_L\right)}}. $$

All three of the free parameters in the model were fit using maximum likelihood estimation (Holt & Laury, 2002; Luce, 1959).

### Results

**Fig. 2:**
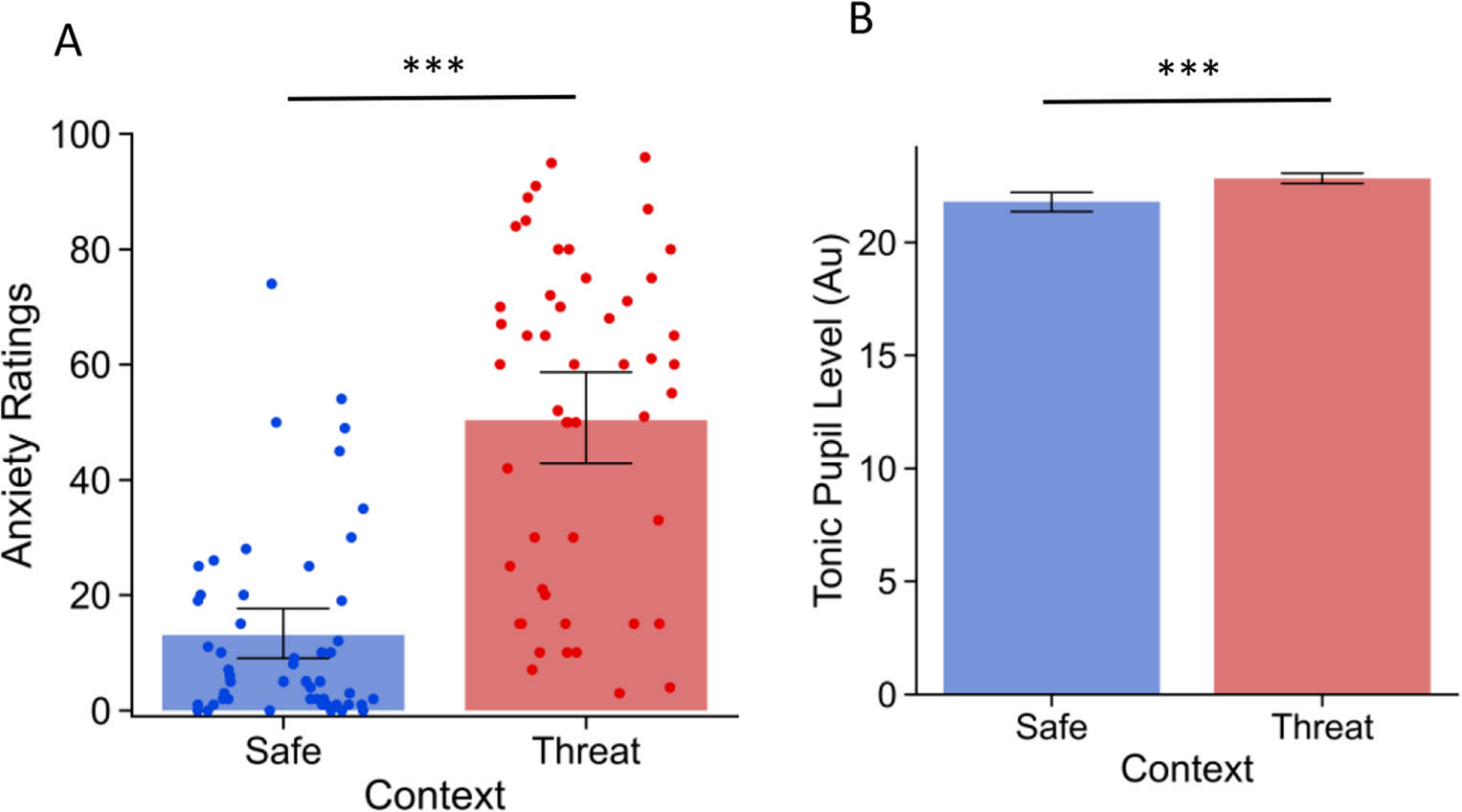
Manipulation check with subjective rating (**A**) and physiology (**B**) for ToS experiment. Error bars represent 95% confidence intervals. Note. *** *p* < .001

Participants’ subjective reports indicated that the threat of shock manipulation was effective in eliciting arousal. Participants indicated they were more anxious during the threat blocks (*M* = 50.37, *SD* = 28.57) compared to the safe blocks (*M* = 13.06, *SD* = 16.83), *t*(50) = 11.078, *p <* .001, *d* = 1.485 95% CI [1.099, 1.871] (see Figure 2a).

In addition to subjective reports, we also measured pupil dilation as an independent physiological proxy for arousal. We ran a multilevel linear model (MLM) to assess trial by trial variation in physiological arousal. Consistent with the subjective reports, pupil dilation indicated that the threat of shock manipulation was successful at inducing arousal. Baseline, pre-trial pupil was more dilated under threat compared to the safe context, *t*(7,984) = 6.892, *p* < .001, *b* = 1.127, 95% CI [0.807, 1.448] (see Figure 2b).

#### Risk and Ambiguity Attitudes

**Fig. 3:**
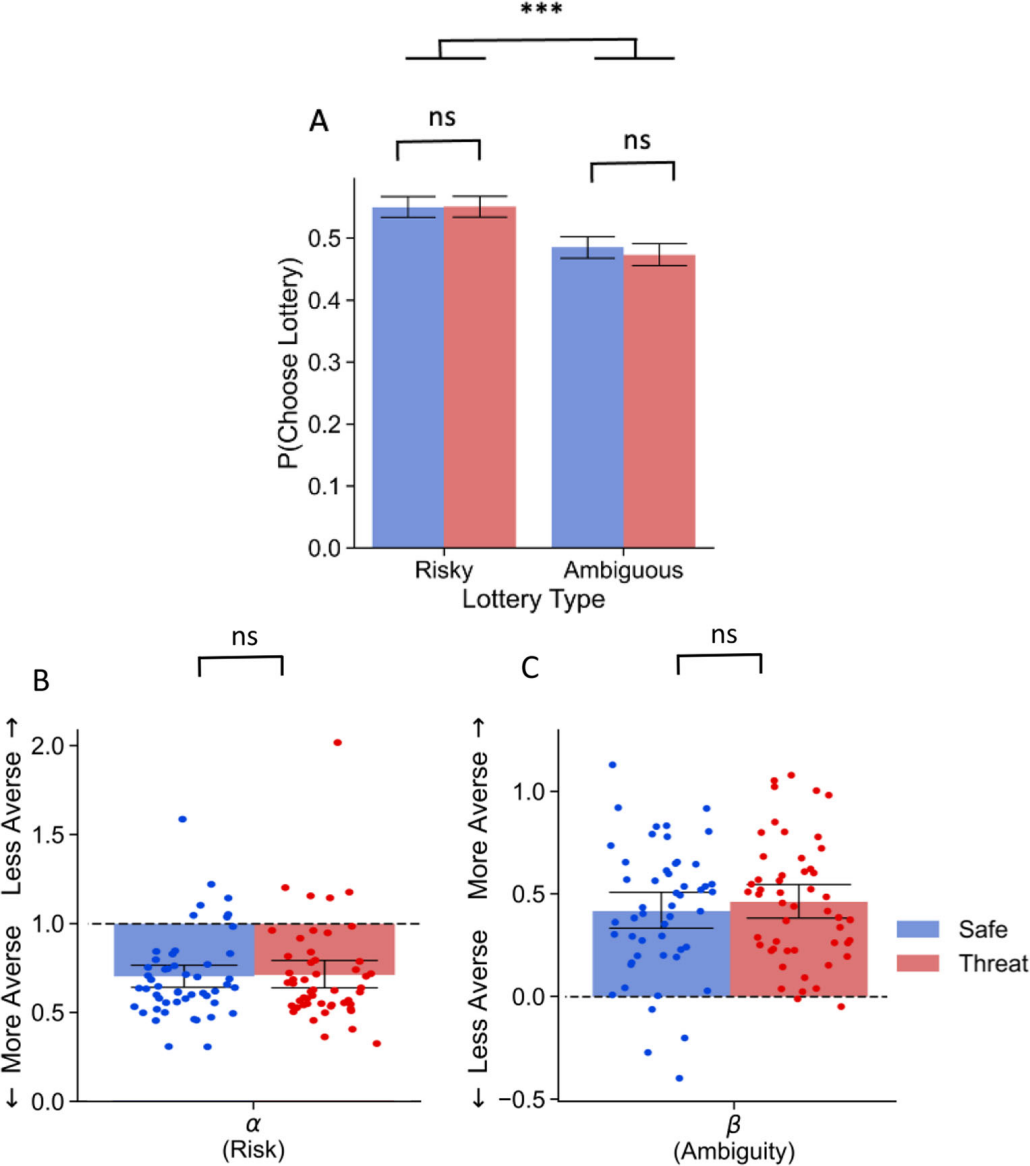
Choice probability across lottery types and context (**A**). Parameter estimates separated by context (**B**) for ToS experiment. Dotted lines represent the neutral point for the given uncertainty type. Error bars represent 95% confidence intervals. Note. *** *p* < .001

We evaluated uncertainty attitudes with a non-parametric analysis of the proportion of times participants chose the uncertain lottery over the guaranteed option. In line with previous work (Ellsberg, 1961; Levy et al., 2010; FeldmanHall et al., 2016; Raio et al., 2021), participant choice data revealed evidence for ambiguity aversion. Despite the fact that the risky and ambiguous lotteries had the same expected values, participants were 1.32 times more likely to choose the lottery when it was risky compared to ambiguous, *z* = 5.517, *b* = 0.279, *p* > .001, *OR* = 1.322 95% CI [1.197 1.459]. However, there was no main effect of block type, *z* = −0.980, *b* = −0.049, *p* = .327, *OR* = 0.952 95% CI [−0.238 0.111], nor was there a block type by lottery type interaction, *z* = 0.789, *b* = 0.056, *p* = .430, *OR* = 1.058 95% CI [−0.084 0.196] when assessing lottery choices (see Figure 3a).

Our experimental design allowed us to implement a computational model, which offers a more sensitive, individual-specific estimate of participants’ attitudes toward ambiguity (*β*) and risk (*α*) and, therefore, may be more sensitive to small effects not captured by lottery choice data alone. Consistent with the literature and our lottery choice data, participants’ parameter estimates suggested strong ambiguity aversion (*M*_*β*_ = .478, *SD*_*β*_ = .329) as indicated by a value greater than zero. Similarly, participants were averse to risk (*M*_*α*_ = 0.720, *SD*_*α*_ = .250) indicated by a value less than one. As hypothesized, participants were no more risk averse under threat (*M*_*α*_threat_ = 0.72, *SD*_*α*_threat_ = .27) compared to a safe context (*M*_*α*_safe_ = 0.72, *SD*_*α*_safe_ = .23), *t*(51) = −.524, *p* = .603, *d* = −.023 95% CI [−.412, .366]. In addition, consistent with our previous lottery choice analysis, there was no evidence that participants ambiguity aversion changed between the threat (*M*_*β*_threat_ = 0.501, *SD*_*β*_threat_ = 0.314) or safe context (*M*_*β*_safe_ = 0.45, *SD*_*β*_safe_ = .344), *t*(51) = −1.592, *p* = .117, *d* = −.103 95% CI [−.493, .286] (see Figure 3b).

#### Bayesian Estimation

Due to the inability to confirm the null hypothesis with frequentist statistics, we employed Bayesian estimation to obtain the likelihood ratio between the null and alternative hypotheses for the two contexts. This was done separately for risk and ambiguity parameters. After subtracting the threat parameters from those under the safe context we estimated the effect size. Specifically, we evaluated whether the difference vector was mostly likely centered on (i.e., their attitudes *did not* change across contexts; the null hypothesis) or not centered on zero (i.e., their attitudes *did* change across contexts; the alternative hypothesis). The effect size under the null hypothesis was estimated with a normal distribution centered at zero with a non-informative, Jeffery’s prior distribution estimating the variance of the effect. For the alternative hypothesis, we used the same Jeffery’s prior for the variance of the effect. We estimated the mean of the effect size for the alternative prior with a Cauchy’s distribution with mean zero and scale factor $$ \raisebox{1ex}{$1$}\!\left/ \!\raisebox{-1ex}{$\sqrt{2}$}\right. $$. The Bayes factor is the ratio of the two likelihood functions and provides a metric for how likely the data would be produced under the two hypotheses. Numbers greater than 1 indicate the data was more likely produced by the alternative model, while numbers less than one provide more evidence in favor of the null hypothesis. The risk and ambiguity parameters both yielded Bayes factors under 1 (*BF*_*α*_*=* 0 .176, *BF*_*β*_ = 0 .528) indicating the data were nearly 5.7 times more likely under the null hypothesis compared to the alternative for the risk parameter and roughly 1.9 times more likely under null than the alternative for the ambiguity parameter.

#### Questionnaires

Several questionnaires were included to assess subjective reports of perceived stress, anxiety, and intolerance of uncertainty. Unexpectedly, intolerance of uncertainty was positively correlated with the raw probability of choosing the ambiguous option in both the safe, *r*(48) = .29, *p* = .044, and threat context, *r*(48) = .31, *p* = .030. As participants scored higher on intolerance of uncertainty, they were also more likely to choose the ambiguous lottery. While state anxiety was not related to either choice probability, trait anxiety was related to both, *r*_safe_(48) = .31, *p* = .033, *r*_threat_(48) = .30, *p* = .034. Perceived stress was also not related to either choice probability.

Surprisingly, the intolerance of uncertainty scale was not correlated with estimates of ambiguity attitudes derived from the computational model (*p*’s > .19). Trait anxiety was correlated to ambiguity estimates under safe, *r*(48) = −.32, *p* = .022, but not threat contexts, *r*(48) = −.20, *p* = .164. Similar to the raw probability data perceived stress was not related to either parameter estimate (*p*’s > .26).

### Conclusion

Consistent with the previous literature (Ellsberg, 1961; Levy et al., 2010; FeldmanHall et al., 2016; Raio et al., 2021), while participants were averse to both types of uncertainty, they were relatively more averse to ambiguity. We implemented a threat of shock design and confirmed its efficacy through subjective ratings as well as pre-trial pupil dilation. Although we did not specifically measure α-amylase, this design has been shown to reliably stimulate a SAM response (Maruyama et al., 2012). As expected, we found no evidence that changes in physiological arousal incidental to the choice affected preferences toward risk. However, in contrast to studies showing a correlation between arousal integral to the choice and ambiguity attitudes (FeldmanHall et al., 2016), we found no evidence that physiological arousal incidental to the choice affects ambiguity preferences. These null results were confirmed using Bayesian statistical analyses.

## Experiment 2: TSST

### Method

#### Participants

Fifty-six participants were recruited to participate in a psychosocial stress study. Participants were excluded prior to participation if they were not fluent in English, had a recent history of mental illness, had ongoing hormone therapy, or had participated in a study involving mild acute stress in the previous 12 months. Four participants were excluded due to technical difficulties, one participant disclosed hormone replacement after the study concluded and was excluded, one participant decided to withdraw their participation during the stress manipulation, and finally three participants were excluded due to poor model fit (outlier cases of pseudo *R*^2^). A total of 48 participants were included in analyses (*M*_Age_ = 23.3, *SD*_Age_ = 5.61) split across the control (*N* = 26) and the stress condition (*N* = 22).

#### Procedures

Participants were randomly assigned to either the stress or control condition and completed a lottery task akin to the 120 trials from the safe context in Study 1. Upon arrival, participants viewed a 15-min low arousal documentary about trains to stabilize their baseline affective states. The lottery task instructions were given before the stress or control manipulation to avoid the potential confound of stress affecting instruction comprehension as well as to minimize the time between stress induction and task onset. After participants were given the instructions for the lottery task, participants were taken to a separate room for the stress manipulation (see below). The lottery task was conducted in a third experiment room. Prior to the onset of the lottery task but after the stress manipulation, all participants had a comprehension check, a task reminder, and four practice trials to ensure they remembered and understood the task. After the lottery task was complete, participants completed a series of surveys including: the Positive Affect Negative Affect Scale (PANAS), the State Trait Anxiety Inventory (STAI), and the Intolerance of Uncertainty (IoU) scale. Finally, participants were debriefed about the nature of the lottery task and stress manipulation.

#### Stress Induction

We utilized the Trier Social Stress Test (TSST) to induce acute psychosocial stress in the stress condition. The TSST elicits reliable increases in subjective reports of stress and salivary free cortisol (Maruyama, 2012; Kirschbaum, 1993). Participants were instructed that they will have 10 min to prepare a 5-min presentation on why they would be the perfect candidate for their ideal job. Participants were told that the interview will be recorded and reviewed by a panel of judges. After the 10-min preparation time, two confederate judges entered the room wearing lab coats and holding clipboards. Confederates were taught to refrain from expressing any emotion in their face or body during the manipulation. Following the speech portion of the task, participants started the math portion of the task where they were instructed to count down by 13’s starting with the number 1,022 as quickly as possible. If the participants took too long to respond or responded incorrectly, they were asked to restart from the number 1,022. Adapted from Guez et al. (2016), all stressful elements of the test were removed for the control participants. After the preparation phase participants were left alone in the room for 5 min including during the control speech phase where they were instructed to practice the speech to themselves. Participants never saw any recording devices. After 5 min the researcher returned with scratch paper for participants to perform the math section. They were under no social pressure to work quickly and were told their answers would not be checked. All participants provided retrospective anxiety ratings of each component of the manipulation.

#### Saliva Collection

**Fig. 4:**
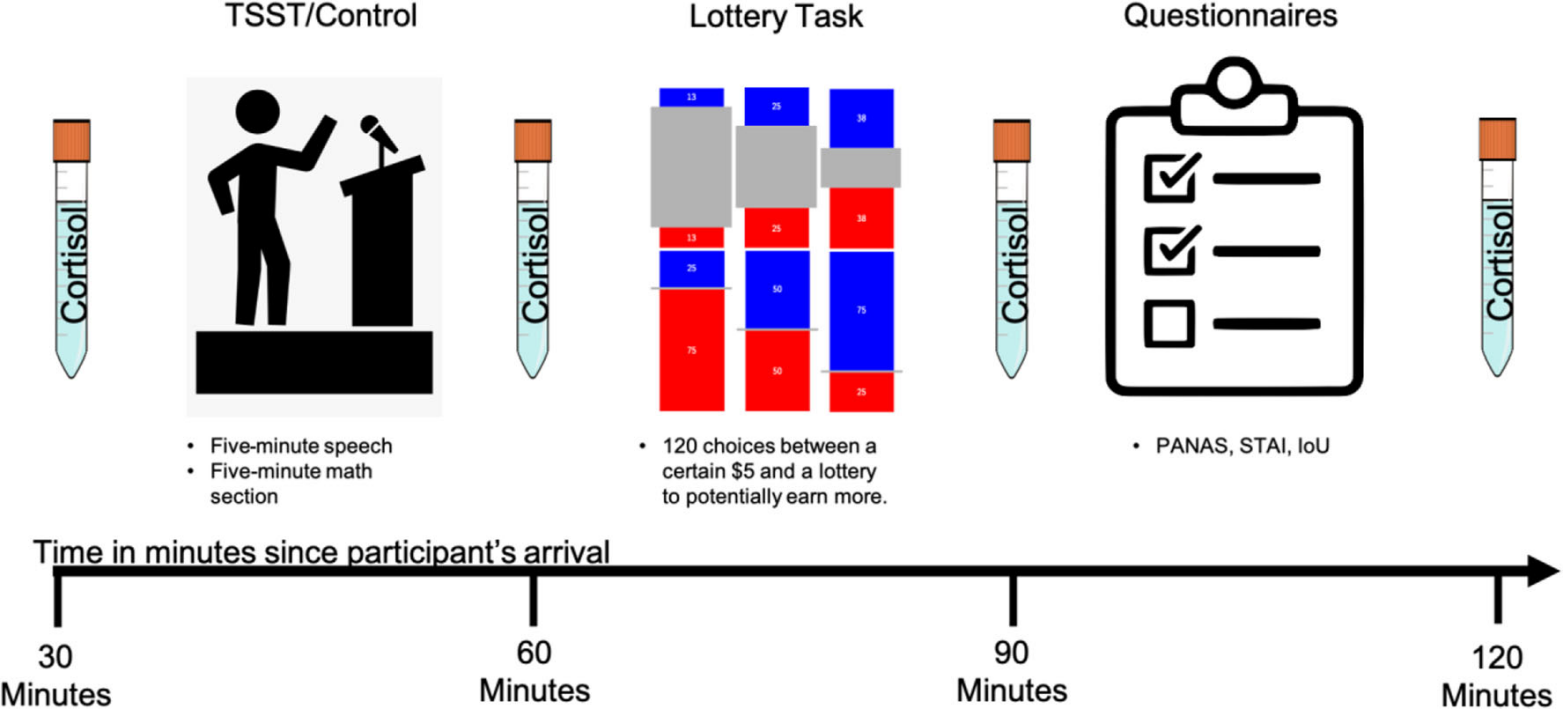
Timeline for TSST study. Participants enter and watch a video to stabilize baseline emotions. After learning the task instructions, we collected their first saliva sample followed by the stress manipulation, sample T1, the lottery task, sample T2, the questionnaires, and finally sample T3

Four times throughout the session, we collected saliva samples as well as subjective anxiety ratings. These saliva collections and subjective ratings occurred at baseline (T0) after both the emotion stabilization video and lottery instructions as well as every 30 min after the baseline time (T1–T3). Assessments T1–T3 corresponded to after the stress manipulation, partly through the lottery task, and finally after the surveys were completed, respectively (see Figure 4). To collect the saliva samples, participants were instructed to place a cotton swab in their mouth for 2 min, ensure that the swab was completely soaked in their saliva in the allotted time, and then place the swab into a sterile tube.

Cortisol data are commonly positively skewed, likely due to the fact that only positive values are attainable and that these data have inherent nonlinearities (Miller et al., 2013). In order to correct this skewness, we log transformed these data as employed in previous studies (Lenow et al., 2017; Lighthall et al., 2013; and Otto et al., 2013). Additionally, in order to obtain a single value for each individual we followed the protocol specified in the literature (Lenow et al., 2017; Lighthall et al., 2013; and Otto et al., 2013) to create a log *cortisol* Δ vector (see below). After log transformation, cortisol samples taken after the stress manipulation were averaged and subtracted from pre-stress levels.

$$ \log cort\ \Delta =\frac{\log {cort}_{t=1}+\log {cort}_{t=2}+\log {cort}_{t=3}}{3}-\log {cort}_{t=0} $$

### Results

**Fig. 5:**
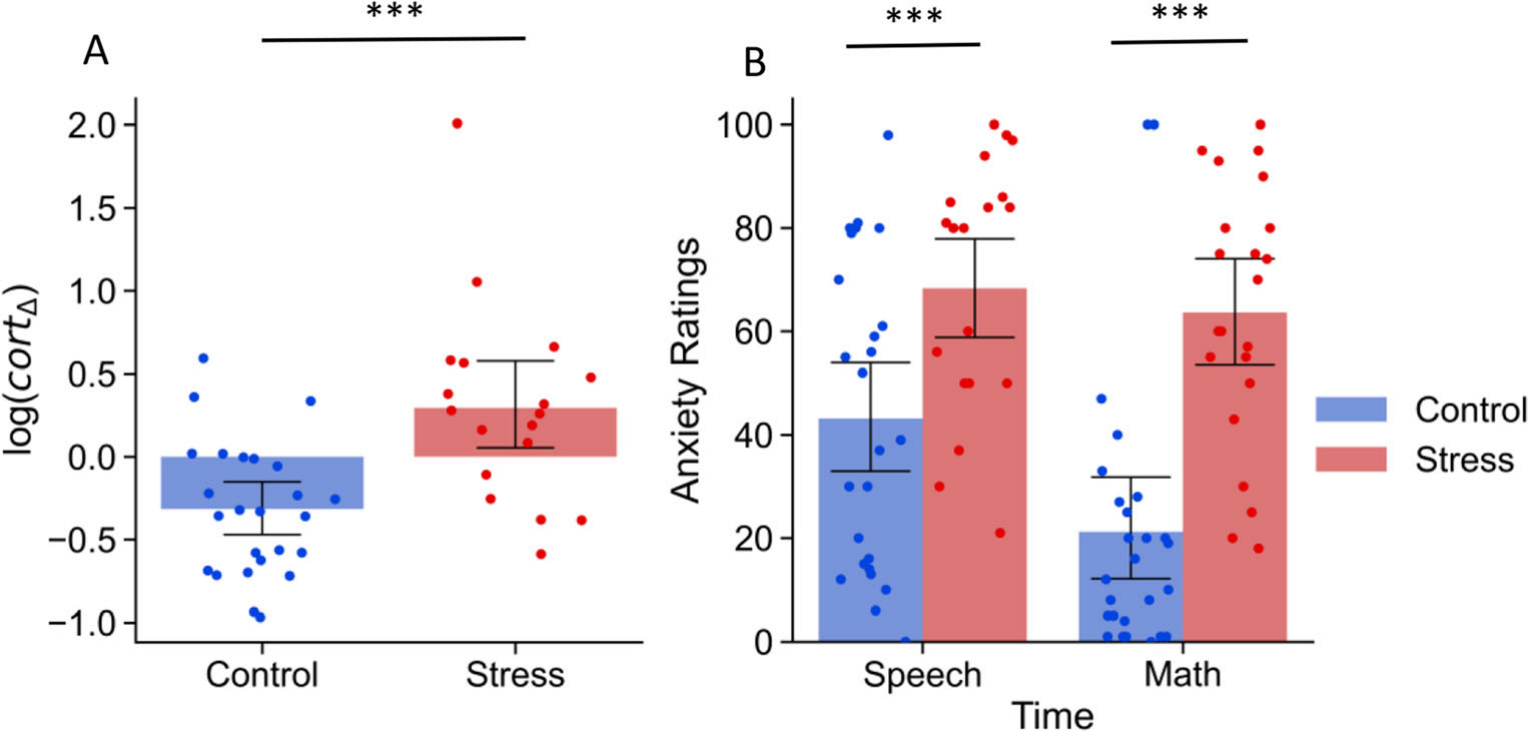
Manipulation check with retrospective anxiety rating (**A**) and log *cort* Δ (**B**) for TSST experiment. Error bars represent 95% confidence intervals. **Note. * *p* < .05, ** *p* < .01, *** *p* < .05. Due to a technical malfunction, cortisol responses for five participants were not included in these analyses

Participants who were in the TSST condition reported being more anxious than those in the control condition in the speech, *t*(46) = 3.307, *p* = .001, *d* = 0.941 95% CI [.32, 1.55], and math portion of the manipulation, *t*(46) = 5.683, *p* < .001, *d* = 1.638 95% CI [0.96, 2.31] (see Figure 5b). These behavioral reports were supported by physiological data. Free salivary cortisol indicated significant elevation of the cortisol response in the stress relative to the control group, *t*(41) = 3.758, *p* < .001, *d* = 1.237 95% CI [0.55, 1.91] (see Figure 5a) indicating the manipulation was successful.

#### Risk and Ambiguity Attitudes

**Fig. 6:**
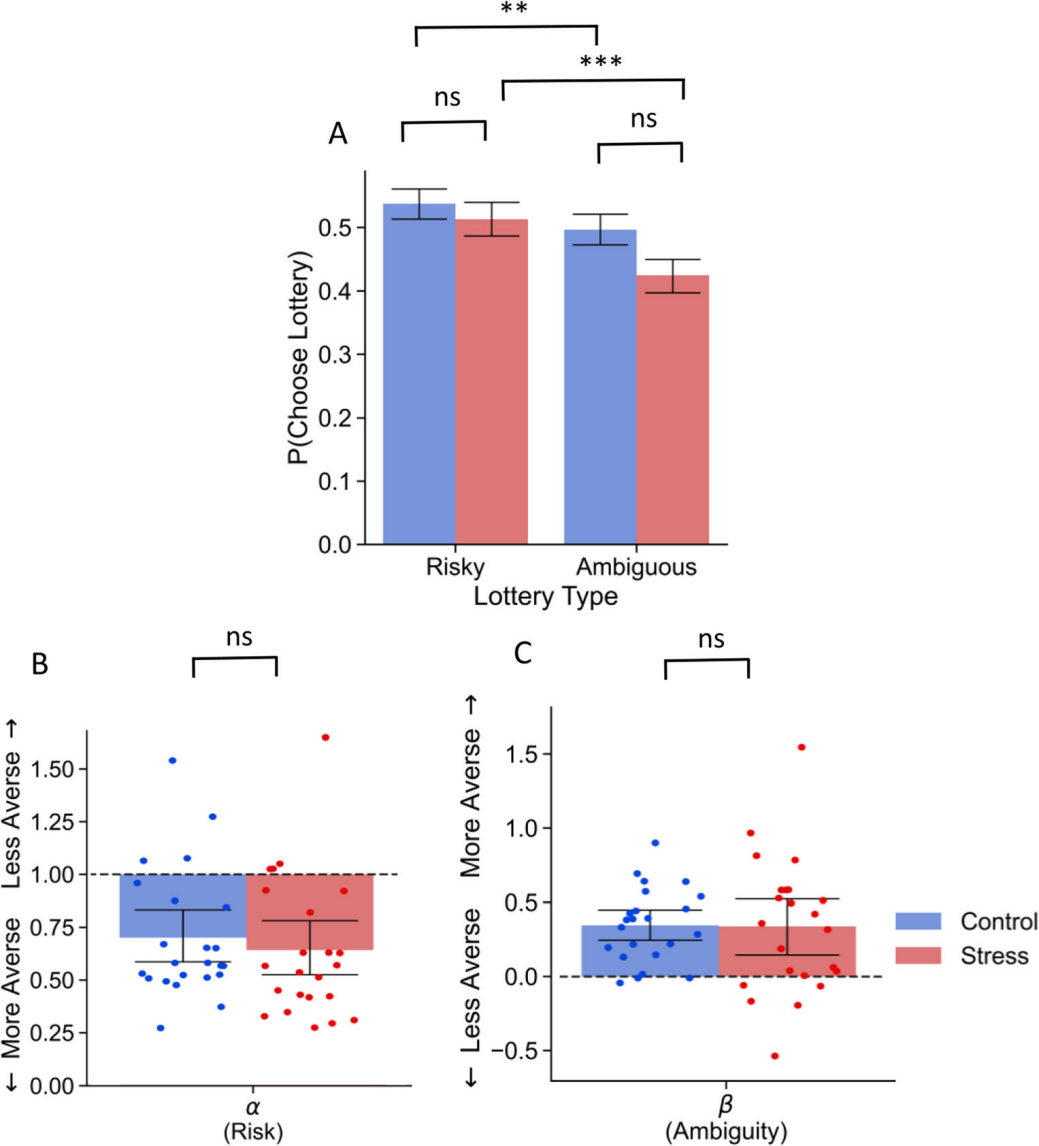
Choice probability across lottery types and context (**A**). Parameter estimates for risk (**B**) and ambiguity (**C**) separated by condition for TSST experiment. Dotted lines represent the neutral point for the given uncertainty type. Error bars represent 95% confidence intervals. Note. ** *p* < .01, *** *p* < .05

As expected, an examination of the choice probabilities for Experiment 2 also yielded evidence for ambiguity aversion. Risky lotteries were about 1.2 times more likely to be chosen compared to ambiguous lotteries, *z* = 2.396, *b* = .0173, *p* = .016, *OR* = 1.188 95% CI [1.03, 1.37]. While there was no main effect for experimental condition on participants’ choice probabilities, *z* = −1.56, *b* = −0.317, *p* = .118, *OR* = 95% CI [0.485, 1.092], unlike in Study 1 there was a significant group by lottery type interaction, *z* = 1.99, *b* = 0.221, *p* = .046, *OR* = 1.246 95% CI [1.004 1.547]. In order to determine the nature of this interaction, we conducted simple effects tests. If this interaction was due to an effect of acute stress on ambiguity attitudes, we would expect the two groups to differ on the probability they chose the ambiguous lotteries, but not the risky lotteries. As expected, there were no group differences for risky lotteries, *z* = −1.438, *b* = −0.367, *p* = .150, *OR* = .693 95% CI [0.415 1.152]. However, there also were no significant group difference for ambiguous lotteries, *z* = −0.574, *b* = −0.099, *p* = .566, *OR* = .904 95% CI [0.638 1.281]. Instead, we found that people in the stress group were relatively more averse to ambiguity compared to risk, *z* = 4.731, *b* = 0.396, *p* < .001, *OR* = 1.486 95% CI [1.261 1.752], and this relative difference appeared to be stronger than the differences in uncertainty preference in the control group, *z* = 2.390, *b* = 0.172, *p* < .01, *OR* = 1.187 95% CI [1.031 1.367] (see Figure 6a). To summarize, the interaction was not driven by group differences in ambiguity aversion, but rather it was driven by the fact that the relative differences in aversion to ambiguity compared to risk were more extreme in the stress group compared to the control group.

When we examined the parameters estimated by the computational model, there was no evidence that stress affected either risk or ambiguity attitudes. On average, participants were averse to ambiguity in both the control (*M*_*β*_ = 0.371, *SD*_*β*_ = 0.274) and stress groups (*M*_*β*_ = 0.373, *SD*_*β*_ = 0.5) indicated by a *β* greater than zero. However, there were no significant differences in ambiguity attitudes between these groups, *t*(45) = .022, *p* = .983, *d* = −.006 95% CI [−.594, .581]. While there was evidence for risk aversion in both groups, there was again no evidence that the level of aversion differed between groups, *t*(45) = 1.186, *p* = .245, *d* = −.341 95% CI [−.251, .933] (see Figure 6b,c).

#### Bayesian Estimation

We implemented the same Bayesian analysis in ToS experiment to confirm the null hypothesis in the TSST experiment. We utilized the same distributions for the null and alternative hypotheses as used in Experiment 1. Consistent with the Experiment 1, risk and ambiguity parameters both yielded Bayes factors under 1 (*BF*_*α*_*=* 0 .503, *BF*_*β*_ = 0 .289). These Bayes factor values indicate that it is nearly twice as likely that there are no differences between groups on risk attitudes and nearly 3.5 times more likely that there are no differences between groups on ambiguity attitudes.

#### Questionnaires

As in the ToS experiment, we ran a series of correlations between the questionnaires and participants’ parameter estimates and choice responses. Surprisingly pooling across subjects, Intolerance of Uncertainty was neither correlated with ambiguous choice probabilities nor ambiguous preference estimates (*p*’s < .14). Similarly, state (*p*’s < .52) and trait (*p*’s < .13) anxiety ratings were unrelated to choice probabilities or model estimated uncertainty preferences. Finally, perceived stress was again unrelated to either metric (*p*’s < .53).

## General Discussion

In this study, we explored the relationship between physiological arousal, stress, and decisions under uncertainty. Consistent with previous research (Ellsberg, 1961; Levy et al., 2010; FeldmanHall et al., 2016; Raio et al., 2021), we found that people are averse to both risk and ambiguity but are relatively more averse to ambiguity. Additionally, as predicted we found that incidental physiological arousal and acute psychosocial stress have no impact on risk preferences. However, in contrast to our hypotheses, we found no evidence that either incidental affective state altered people’s preferences toward ambiguity. Raw choice data was supplemented by a computational model for uncertainty attitudes which yielded results consistent with the choice data. Finally, in both experiments we followed these results up with Bayesian analyses to affirm the null hypothesis.

Previous research found a significant relationship between integral physiological arousal and ambiguity preferences, but not risk preferences (FeldmanHall et al., 2016). Based on this result, we hypothesized that physiological arousal might serve as an affective signal about the quality of ambiguous options. Furthermore, we hypothesized that if we manipulated physiological arousal incidental to the choice, this arousal signal might be nonoptimally integrated into the choice option’s subjective valuation. Despite integral arousal correlating with ambiguous choices, physiological arousal incidental to the choice yielded no such relationship. These data indicate that decisions under uncertainty are resilient to mild, transient, incidental affective influences, both for considerations of risk and ambiguity. Therefore, it seems that while arousal does contribute to ambiguity aversion, the arousal response must be driven by the choice itself. Transient arousal experienced near the decision does not appear to get integrated into the choice valuation. Consistent with this hypothesis, Baillon et al. (2018) found that under time pressure ambiguity attitudes did not change. However, participants’ sensitivity to the amount of ambiguity was reduced. Although ambiguity insensitivity was not intentionally measured in this study, participants’ insensitivity may have been affected by the experimental stressor.

It has been shown that lifetime stress predicts ambiguity preferences (Raio et al., 2021). We hypothesized that if acute stress also affects ambiguity preferences, then the stress group would be relatively more averse to ambiguity compared to the control group. However, our data suggest that acute stress does not affect uncertainty preferences. While we had a significant interaction in the hypothesized direction for the choice probabilities, follow-up analyses suggested that the primary driver was not based on individuals in the stress condition being more averse to ambiguity compared to the control participants. Furthermore, model-derived attitudes did not show any significant interactions, and these results were followed up with Bayesian analyses which indicated higher evidence for the null hypothesis. We conclude that an acute stress manipulation does not impact ambiguity preferences.

It has been suggested that lifetime stress, especially early life adversity, shapes the way ambiguity is assessed because it results in a prior for expected outcomes in uncertain situations in the future (Raio et al., 2021). Stressful events early in life may teach people to expect the worst outcome in the face of uncertainty. Although it is often suggested that ambiguity aversion is not optimal (Ellsberg, 1961), a life history of many stressful events might bias one toward the conclusion that negative outcomes are more likely in ambiguous contexts, and, in this case, the optimal choice is to avoid ambiguity if possible.

In contrast to lifetime stress, the current study found acute stress does not result in a similar bias to avoid ambiguity. However, acute stress has been shown to affect a variety of other types of choices. For example, one study had participants complete a series of temporal discounting choices before and after the TSST stress and found that stress increased discount rates for subjects who exhibited an increase in cortisol in response to the manipulation (Kimura et al., 2013). Another study found that stress led people to be more willing to engage in harmful actions for betterment of the group compared to control participants for personal moral choices (Youssef et al., 2012). Previous research on acute stress and risk has yielded mixed results. Although some studies have shown that acute stress increases risky decision making (e.g., Pabst et al., 2013), when one separates out the various decision factors (e.g., loss aversion, risk aversion, and increased noise) acute stress appears to have no impact on risky choices (Sokol-Hessner, 2016). To our knowledge there is only one published study that explored acute stress and ambiguity preferences (Buckert et al., 2014) that found acute stress did not impact ambiguity aversion in a lottery task. Similarly, our data suggest that a single mild stress manipulation and the corresponding affective changes that are elicited are not incorporated into the decision valuation.

As research on the impact of emotion on decision making has grown, it is increasingly apparent that their interaction is often nuanced. These current results highlight the fact that affect is a multifaceted phenomenon, and one affective component can selectively affect one type of decision in some circumstances and not others. As we continue to uncover the influence of affect on decision making it is important to specifically characterize which particular affective process interacts with which types of decisions and under what conditions.

## Additional Information

### Availability of data and material

Data from this work are available here https://osf.io/n6em2/
